# Sinomenine regulates immune cell subsets: Potential neuro-immune intervene for precise treatment of chronic pain

**DOI:** 10.3389/fcell.2022.1041006

**Published:** 2022-12-22

**Authors:** Wei-Dong Lai, Song Wang, Wen-Ting You, Si-Jia Chen, Jun-Jun Wen, Cun-Rui Yuan, Meng-Jia Zheng, Yan Jin, Jie Yu, Cheng-Ping Wen

**Affiliations:** ^1^ School of Basic Medical Science, Zhejiang Chinese Medical University, Hangzhou, China; ^2^ Department of Pharmacy, The Affiliated Wenling Hospital of Wenzhou Medical University, Wenling, China; ^3^ Xinhua Hospital of Zhejiang Province, The Second Affiliated Hospital of Zhejiang Chinese Medical University, Hangzhou, China

**Keywords:** sinomenine, sensory neurons, immune cells, glial cells, chronic pain

## Abstract

Chronic pain is a disease of long-lasting pain with unpleasant feelings mediated by central and (or) peripheral sensitization, its duration usually lasts more than 3 months or longer than the expected recovery time. The patients with chronic pain are manifested with enhanced sensitivity to noxious and non-noxious stimuli. Due to an incomplete understanding of the mechanisms, patients are commonly insensitive to the treatment of first line analgesic medicine in clinic. Thus, the exploration of non-opioid-dependent analgesia are needed. Recent studies have shown that “sinomenine,” the main active ingredient in the natural plant “*sinomenium acutum* (Thunb.) Rehd. Et Wils,” has a powerful inhibitory effect on chronic pain, but its underlying mechanism still needs to be further elucidated. A growing number of studies have shown that various immune cells such as T cells, B cells, macrophages, astrocytes and microglia, accompanied with the relative inflammatory factors and neuropeptides, are involved in the pathogenesis of chronic pain. Notably, the interaction of the immune system and sensory neurons is essential for the development of central and (or) peripheral sensitization, as well as the progression and maintenance of chronic pain. Based on the effects of sinomenine on immune cells and their subsets, this review mainly focused on describing the potential analgesic effects of sinomenine, with rationality of regulating the neuroimmune interaction.

## 1 Introduction

According to the IASP, “chronic pain” is an unpleasant emotional experience with its pain sensation lasting longer than 3 months or the expected time for disease recovery (Knotkova et al., 2021). Recently, an epidemiological study shows that more than 20% of U.S. adults experience chronic pain (Yong et al., 2022). Long-term pain induces a multitude of harmful effects like anxiety, insomnia, depression, premature aging and other harmful symptoms, seriously threatens the physical and mental health of patients (Finan et al., 2013; Rachor and Penney 2020; Lahav et al., 2021; Ma et al., 2021). Thus, chronic pain has been gradually recognized as a disease with biological, social, psychological, and spiritual manifestations, and is vulnerable to many risks, such as smoking, alcohol consumption, exercise, nutrition, and medical intervention (Paller et al., 2009; Andersson et al., 2013; Mills et al., 2019). However, the pathogenesis of chronic pain has not been fully clarified yet (Fitzcharles et al., 2021).

Immune cells and central glial cells have been shown to contribute to the development and maintenance of chronic pain through neuro-immune interactions (Grace et al., 2014; Sanmarco et al., 2021). For example, the sensory neurons release neuron-derived mediators, such as adenosine triphosphate (ATP), neuron-peptides and macrophage-colony stimulating factor (M-CSF), to simulate the polarization of macrophages (Merighi et al., 2008; Stösser et al., 2011; Burnstock 2017). On the other hand, by secreting different kinds of mediators such as tumor necrosis factor alpha (TNF-α), nerve growth factor (NGF) and interleukin-1beta (IL-1β), some peripheral immune cells, such as macrophages, Schwann cells, lymphocytes and mast cells, enhance the excitability and sensibility of the primary sensory neurons and induce chronic pain, suggesting that interfering the interaction between macrophages and neurons may potentially help to alleviate chronic pain (Shu and Mendell 1999; Lim et al., 2015; Minnone et al., 2017; Aarão et al., 2018; Domoto et al., 2021). In addition, T cells, infiltrating into the dorsal root ganglion (DRG) of nerve injury model animals, were also reported to release leukocyte elastase (LE) and induce chronic pain, while this phenomenon could be inhibited by SerpinA3N inhibitor secreted by DRG neurons (Vicuña et al., 2015). Apart from peripheral immune cells, central glial cells including microglia and astrocytes were also proved to interact with neurons to regulate chronic pain, indicating that inhibiting the interaction between central glial cells and sensory neurons might also be a potential pathway for treating chronic pain (Ji et al., 2016).

**FIGURE 1:**
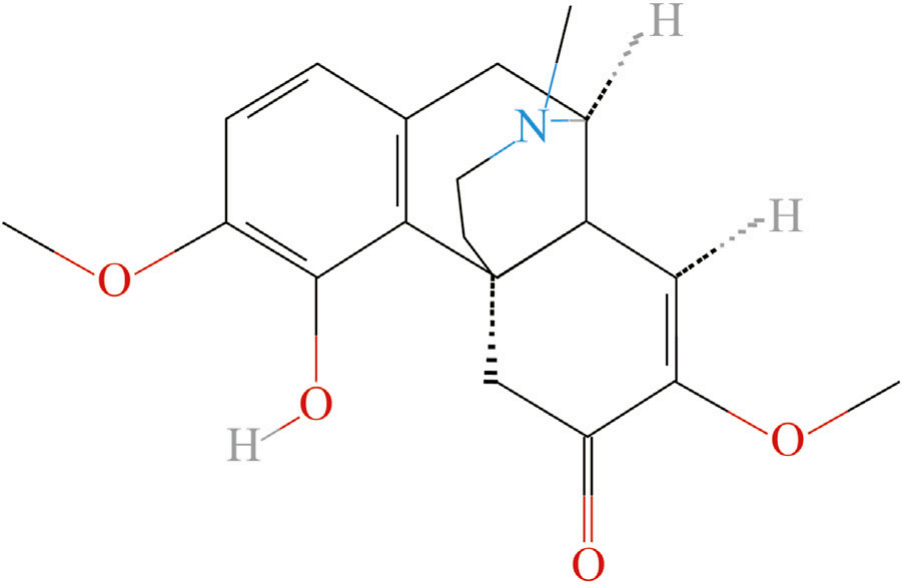
Chemical formula of sinomenine.

It is reported that nearly 20% of chronic pain patients are insensitive to anti-inflammatory and analgesic drugs (Buch 2018; Buch et al., 2021). However, using opioids as an analgesic may have the risks of inducing hyperalgesia, drug resistance and addiction, etc. (Benyamin et al., 2008; Mercadante et al., 2019). Natural plants have been widely used medicinally for centuries in different countries, and many pharmaceutical active compounds derived from natural plant products have been proved to be effective in the treatment of chronic pain (Jiang et al., 2022). We recently screened out the herb, “Qingfengteng” (“*Sinomenium acutum* (Thunb.) Rehd. Et Wils.”) as a frequently prescribed herb for treatment arthritic pain in clinic (Lai et al., 2022). As the main active ingredient of “Qingfengteng,” sinomenine was reported with various biological activities, such as antioxidant, neuroprotection, and antidepressant, etc. (Liu S. et al., 2018; Hong et al., 2022). According to the previous pharmacological studies, sinomenine, also known as “cucoline,” is an alkaloid found in the root of the climbing plant *Sinomenium acutum*, and has a powerful analgesic and anti-tumor effects in clinic (Figure 1). Single administration of sinomenine could significantly alleviate mechanical allodynia in rats with cancer bone pain, while long-term treatment of sinomenine could significantly reduce the neuropathic pain of spinal nerve ligation (SNL) rats and inhibit central sensitization (Chen et al., 2018; Wang X. et al., 2021). As a dextrorotatory morphinan analog with a chemical structure similar to morphine (Figure 1), sinomenine could combine to and activate μ-opioid receptors to exert its central analgesic effect directly (Wang et al., 2008). In addition, a parallel randomized controlled study involving 120 patients showed that sinomenine could be used to assist the clinical first-line drug use, improve its toxic and side effects, reduce the risk of adverse event (AE), and help patients to receive a long course of treatment (Liu S. et al., 2018; Huang et al., 2019; Hong et al., 2022). Thus, it is widely used in the treatment of rheumatoid arthritis, enteritis, ischemia and reperfusion injury, organ allotransplantation and other diseases in clinic.

However, the underlying mechanisms of analgesic effects of sinomenine have not been fully elucidated. This review is aimed to provide researchers with insights into the possible effects of sinomenine on the neuro-immune interaction, through which might be potentially promising to relieve chronic pain, and provides new ideas for the further clinical alternative treatment and pharmaceutical research.

## 2 The effects of sinomenine on peripheral immune cells

### 2.1 Sinomenine regulates macrophage functions in peripheral sensitization

Macrophage derives from embryonic development (tissue resident macrophages) or bone marrow-derived circulating monocyte macrophages (non-tissue resident macrophages). It is one of the most important immune cells with multiple effects, including antigen presentation and phagocytosis effects during the human defensive reaction. Recently, several studies have shown that dysfunction of macrophages is closely related to chronic pain, as well as the development of peripheral sensitization, and the regulation of macrophages is a promising way to treat chronic pain (Gentek et al., 2014; Ginhoux and Guilliams 2016; Domoto et al., 2021). At the site of peripheral nerve injury, the occurrence of endothelial activation results in recruitment of monocytes/macrophages, which sensitized nociceptive neurons and induced peripheral sensitization. Moreover, the phenomenon that macrophages infiltrated into DRG was observed in various models of neuropathic pain, while using chlorophosphate to deplete macrophages helps to alleviate pain, suggesting a crucial role of DRG macrophages in chronic pain (Zhang et al., 2016; Yu et al., 2020). We speculate that the macrophage might be one of the targets for sinomenine, and the potential mechanisms are reviewed as follows:

#### 2.1.1 Sinomenine inhibits inflammatory cytokines secreted from macrophages

**FIGURE 2:**
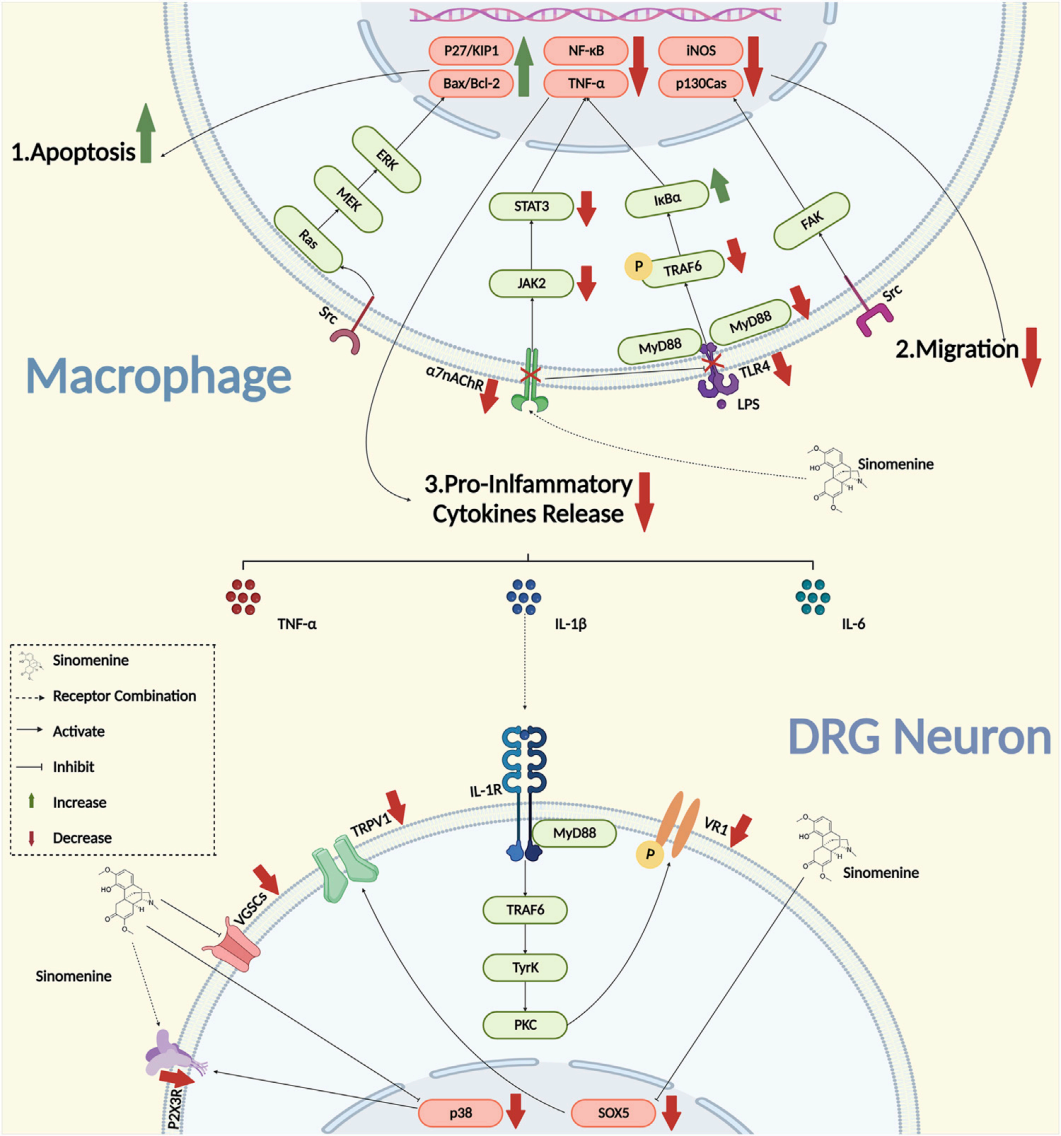
The interaction between macrophages and neurons under the intervention of sinomenine.

Inflammation is the key pathogenesis in the development and maintenance of chronic pain. When the peptidergic nerve fibre was exposed to IL-1 (Interleukin-1), IL-6 (Interleukin-6), and TNF-α, the hyperalgesia that manifested in adjuvant-induced arthritis (AIA) model animals could be induced directly (Lopes et al., 2020). By bounding to the receptors that expressed on DRG neurons, pro-inflammatory cytokines could activate the downstream signaling pathway and upregulate the activities of neurons. However, when the transcription, expression, and the secretion of pro-inflammatory cytokines were inhibited, the chronic pain could be successfully suppressed (Martinez et al., 2008; Schaible 2014; Cook et al., 2018). Sinomenine was proved to have a powerful inhibitory effect on the secretion of various pro-inflammatory cytokines, such as IL-1β, TNF-α, and IL-6 in various diseases (Zhao et al., 2013; Xiong et al., 2017; Kim et al., 2018; Zhang et al., 2018; Wang et al., 2020). It was reported that the concentration of IL-6 and IL-1β were significantly reduced in the serum of collagen-induced arthritis (CIA) rats treated with sinomenine, as well as their foot swelling, serological markers and arthritic scores, suggesting that sinomenine would be a potential drug for the treatment of RA inflammation (Zhou et al., 2008). Yan et al. found that the mRNA expression of TNF-α, IL-1β and IL-6 were decreased, and the mechanical and heat hyperalgesia were both reversed by sinomenine administration in complete Freund’s adjuvant (CFA) model rats (Yuan et al., 2018). In addition, in the rat model of AIA, the secretion of TNF-α, IL-6 and IL-1β were also inhibited by sinomenine treatment (Lan et al., 2016). Furthermore, it was reported that the mRNA of TNF-α, IL-1β, NF-κB, and inhibitor of NF-kappa B (IκB) in rat peritoneal macrophages was inhibited by sinomenine treatment in the AIA model (Figure 2) (Wang et al., 2005). These studies indicated that the inhibitory effects of sinomenine on pro-inflammatory cytokines in different models might be partially dependent on its inhibition of macrophages.

It was also found *in vitro* that the intervention of sinomenine in LPS-induced peritoneal primary macrophages activation, and the its release of pro-inflammatory cytokines might be involved the inhibition of TLR4/myeloid differentiation factor 88 (MyD88)/NF-κB signaling pathway (Yin N. et al., 2020; Zeng and Tong 2020). Zhang et al. reported that sinomenine could also suppress the phosphorylation of tumor necrosis factor-associated factor 6 (TRF6), thereby inhibit the activation of downstream NF-/kB that induced by transforming growth factor activated kinase-1 (TAK1), and implement the inhibitory effect on the MAPK signaling pathway (Zhang et al., 2015). Furthermore, the effect of sinomenine might also partially depend on α7 nicotinic acetylcholine receptor (α7nAChR), because sinomenine, as a ligand, could bind to macrophage α7nAChR to inhibit the expression of lipopolysaccharide receptor (CD14)/TLR4, and then activate the downstream JAK2/STAT3 pathway to exert the anti-inflammatory effects (Figure 2) (Yi et al., 2015; Zhu et al., 2019; Xie et al., 2021). However, it is important to note that, in addition to the receptors and ion channels that expressed on the surface of primary sensory neurons, the effects of sinomenine on specialized membrane proteins that with binding functions to pro-inflammatory cytokines remain to be further explored.

#### 2.1.2 Sinomenine inhibits macrophage proliferation

Liu et al. reported that, in CIA model mice, the increased proliferation of macrophages (CD11b^+^, F4/80^+^, CD64^+^) located in the synovial membrane of the joints was reduced by sinomenine treatment. The numbers of macrophages (CD11b^+^, Ly6C^+^, CD43^+^) in the spleen and lymph nodes were also significantly inhibited. In addition, sinomenine reversed the increased proportion of macrophages (CD14^+^, CD16^+^) in peripheral blood monocytes of RA patients (Liu W. et al., 2018). It was also reported that sinomenine administration could activate extracellular signal-regulated kinase (ERK), increase the expression of pro-apoptotic factors Bcl-2 associated X protein (Bax)/B cell lymphoma 2 (Bcl-2) and the Cyclin-dependent kinase (CDK) regulator P27Kip1, and promote the apoptosis of RAW264.7 cells (He et al., 2005). Moreover, the autophagy of peritoneal macrophage was enhanced in cecal puncture ligation mice treated with sinomenine hydrochloride, which was reversed by the autophagy blocker 3-methyladenosine (Jiang et al., 2015). It indicated that sinomenine might be able to inhibit proliferation by promoting autophagy and apoptosis of macrophages. However, it is of great significance to study the effect of sinomenine on the proliferation of macrophages that infiltrated into peptidergic nerve fibers, which were observed in the DRG in varieties of pain models (Bravo-Caparros et al., 2020; Dai et al., 2020; Yu et al., 2020). Clarifying the role of macrophages infiltrating into DRG in the progression of pathogenesis might provide an important theoretical basis for the research of chronic pain. Furthermore, whether sinomenine could inhibit the proliferation of macrophages by regulating the cell cycle progression of macrophages still remains to be further studied.

#### 2.1.3 Sinomenine inhibits macrophage migration and invasion

The migration and invasion of macrophages are important factors that affect the peripheral sensitization and contribute to the progression of chronic pain. Gao et al. reported that the migration of macrophage was significantly inhibited by sinomenine treatment in carrageenan induced inflammatory pain of mice. As performed *in vitro* experiments, they also found that sinomenine inhibited the migration of RAW264.7 cell, the secretion of TNF-α and IL-6, and the expression of inducible nitric oxide synthase (iNOS), P-Tyr416Src and P-Tyr397FAK, suggesting that sinomenine might inhibit macrophage migration through the proto-oncogene tyrosine-protein kinase Src/focal adhesion kinase (FAK) pathway to alleviate chronic pain (Figure 2) (Gao et al., 2021). Cellular structural plasticity is closely related to the cytoskeleton, as well as the participation of micro-tubules (MTs) and actin (F-actin) (Cain et al., 1981; Chiou and Don 2007; Pegoraro et al., 2017). However, whether sinomenine could regulate microstructural changes and the redistribution of microtubules and actin filaments, to affect the remodeling of the cytoskeleton of macrophages and thus change its migration, still remains to be further investigated.

In addition, sinomenine could also inhibit the invasion of macrophages. According to recent studies, sinomenine regulated osteoclast differentiation by inhibiting the prostaglandin E2 (PGE2)-induced osteoprotegerin (OPG)/receptor activator of nuclear factor-kB ligand (RANKL) ratio, which induced by macrophage invasion (He et al., 2014; Zhou B. et al., 2017). Ou et al. found that, when exposed to sinomenine, the secretion and expression of Matrix metalloproteinase 2 (MMP2), Matrix metalloproteinase 9 (MMP9) and extracellular matrix metalloproteinase inducer (CD147) were all down-regulated, reducing the migration and invasion of macrophages (Ou et al., 2009; Ju et al., 2010). However, it is worth noting that all these effects were not validated in patients or animals with chronic pain. Besides, through any other mechanisms, whether sinomenine could inhibit the invasion of macrophages, to either DRG or central nervous system, to alleviate hyperalgesia of chronic pain remains to be further explored. Moreover, whether sinomenine could reduce cartilage erosion and alleviate osteoarthritis (OA) pain through inhibiting the migration and invasion of macrophages in the joints and synovial membrane still needs to be further investigated.

#### 2.1.4 Sinomenine regulates macrophage polarization

**FIGURE 3:**
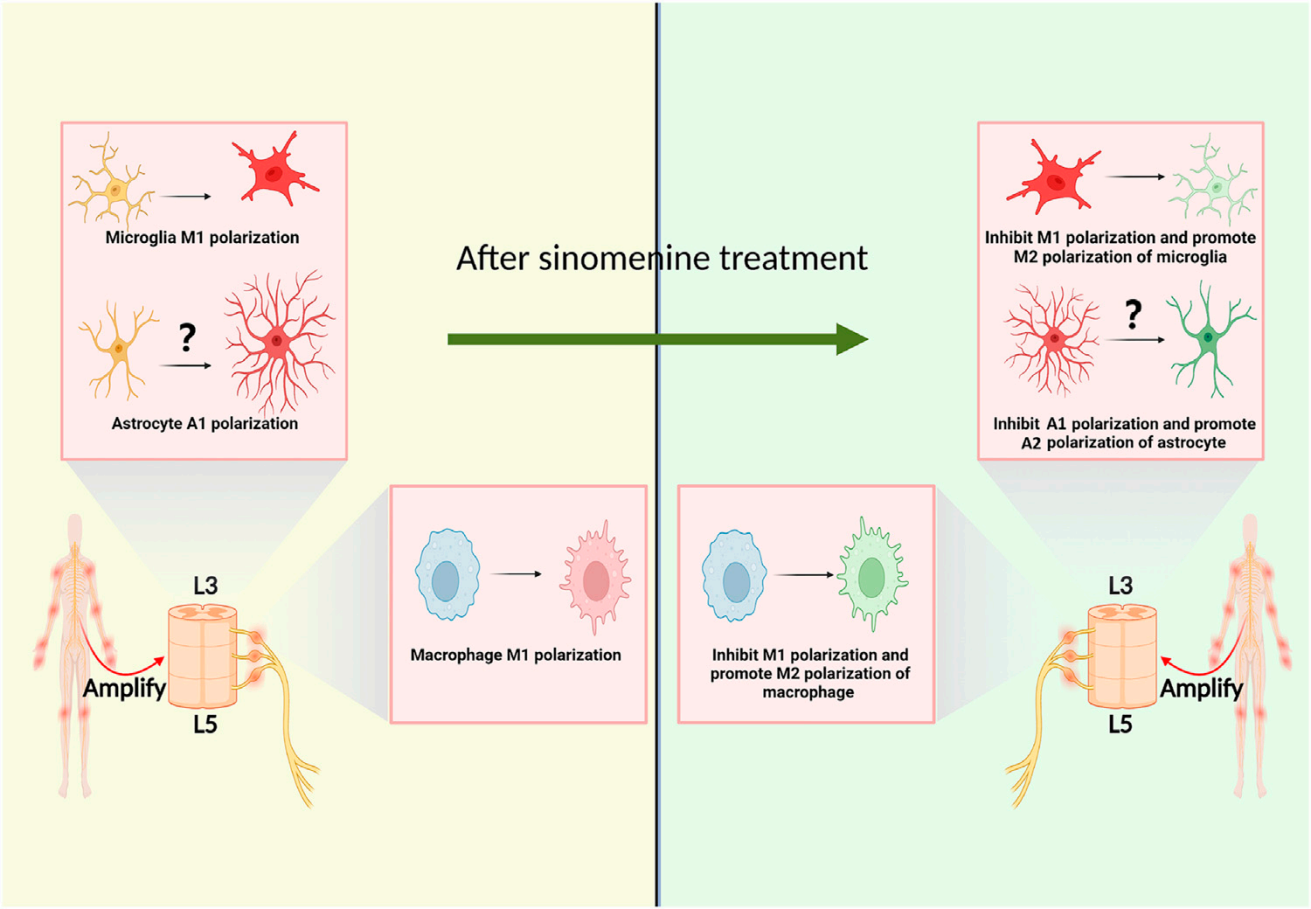
Sinomenine inhibits chronic pain by affecting the polarization of macrophages and glial cells.

Macrophages aggregated into the DRGs after sciatic nerve transection (SNT) in rats, and their classically activated macrophages (M1)/alternatively activated macrophages (M2) polarization ratio was increased (Chen et al., 2022). Ramin et al. reported that M1 macrophage infiltration appeared in the DRG of osteoarthritis pain model mice. The arthritic pain was independent of the tetrodotoxin-resistant voltage-gated sodium channels (Nav1.8 channels) on DRG neurons, but the induction of macrophage M2 polarization could significantly inhibit the pain (Raoof et al., 2021). Furthermore, sinomenine could also inhibit the expression of TNF-α and IL-6, and M1 polarization of peripheral macrophages *in vitro* (Zhi et al., 2022). In the oxygen-glucose deprivation (OGD) experiment, sinomenine could also improve the expression of arginase1 (Arg1) and interleukin-10 (IL-10) of BV2 cells, suggesting that sinomenine might not only has the function of inhibiting macrophages M1 polarization, but also promoting its transition to M2 polarization (Figure 3) (Bi et al., 2021).

### 2.2 Effect of sinomenine on lymphocytes

Lymphocytes play important roles in human adaptive immunity. Several studies showed that dendritic cells (DC) and macrophages could release complements to promote downstream immune activity after antigen presentation, then induce T-helper (Th1) differentiation of CD4^+^ T cells. It’s worth noting that Th1 cells release pro-cytokines to induce inflammatory and chronic pain (Lubbers et al., 2017). The imbalance between Th1/Th2 or T-helper 17 (Th17)/T-regulatory (Treg) cells (Th17/Treg) is an important factor in inducing the body’s immune system disorders. When Th1 and Th17 cells are dominant, the body’s immune system tends to be pro-inflammatory and release large amounts of pro-inflammatory mediators to induce chronic pain. B cells, on the other hand, produce antibodies through differentiated plasma cells, while auto-antibodies could bind to the Fc gamma receptor I (FCγRI) expressed on sensory neurons and induce pain (Qu et al., 2011; Jiang et al., 2017; Wang et al., 2019; Liu F. et al., 2020). Thus, we speculate that lymphocytes may be another target for sinomenine to regulate chronic pain, and the potential mechanisms are reviewed as follows:

#### 2.2.1 Sinomenine inhibits the activation and proliferation of T cells, and the differentiation of B cells

**FIGURE 4:**
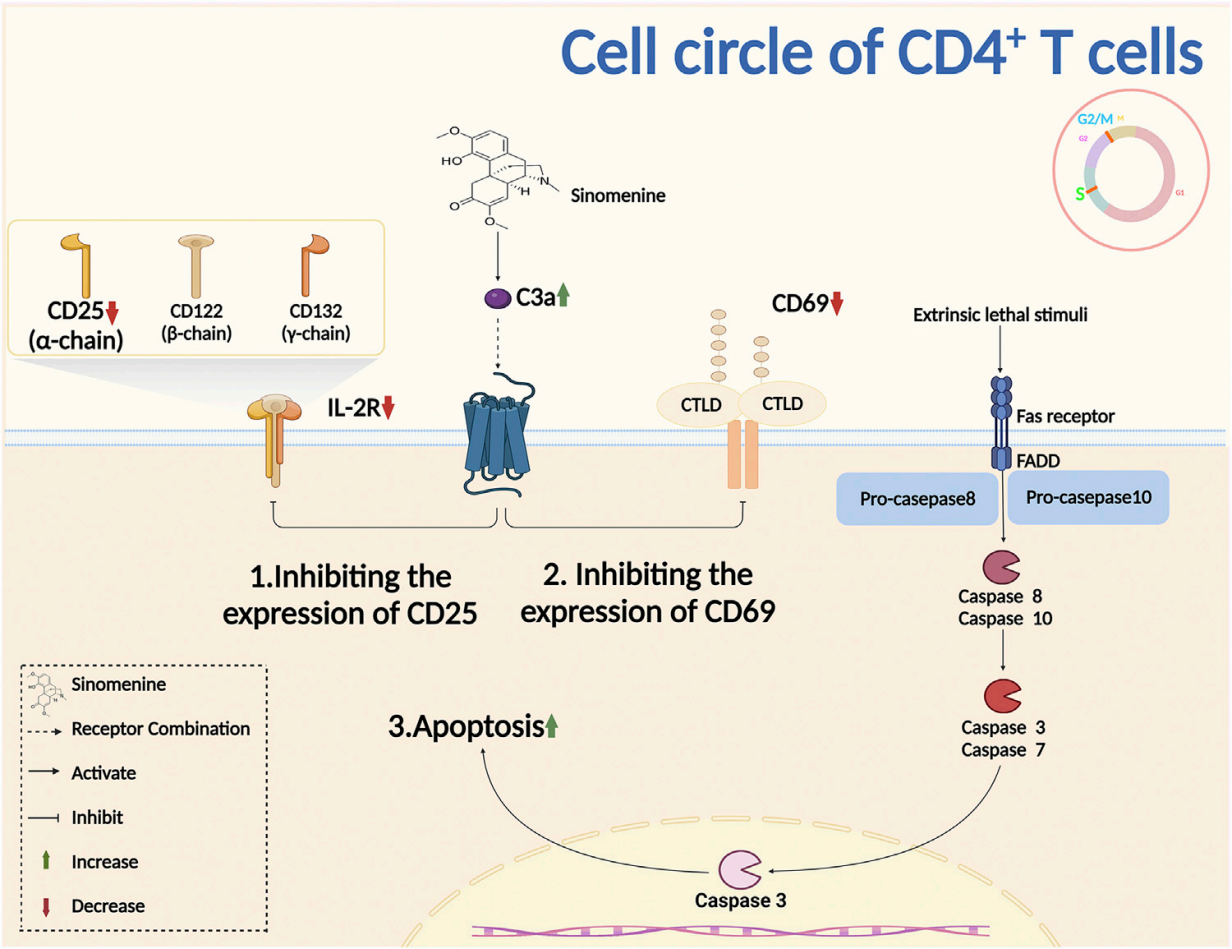
Sinomenine inhibits the proliferation of CD4+T cells.

**FIGURE 5:**
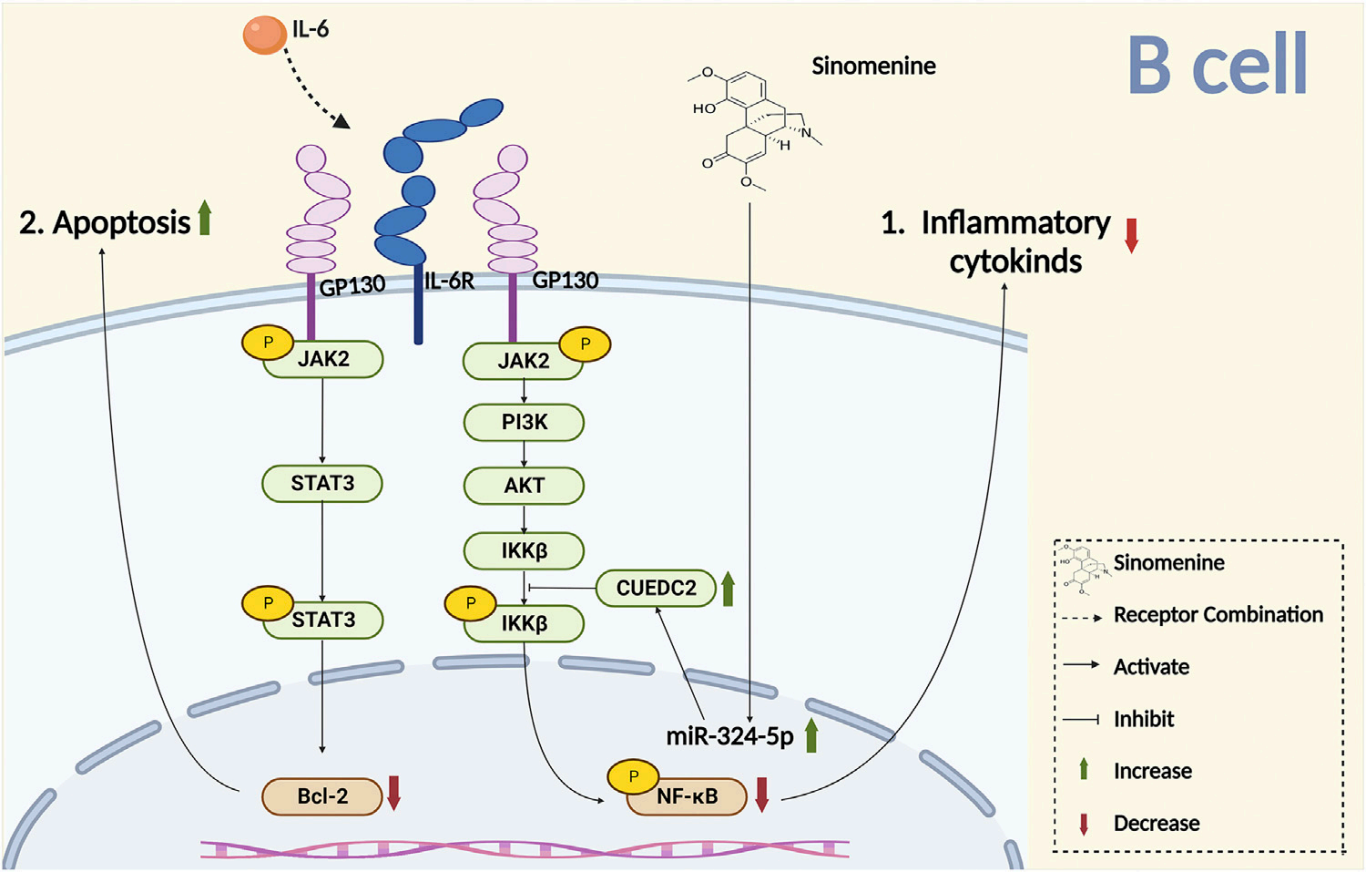
Sinomenine inhibits chronic pain by promoting B cell apoptosis and inhibiting the release of inflammatory cytokines.

Complement is an important component of the innate immune system. It was reported that sinomenine treatment increased the level of plasma complement C3, inhibited Th1 transcription and cytokines secretion, and downregulated the immune response (Cheng et al., 2009). The primary lymphocyte aggregation, and the surface markers CD25 and CD69 of T cell activation were inhibited by sinomenine (Shu et al., 2007). Sinomenine administration also directly affected CD4^+^ T proliferation by blocking the cell cycles, evidenced by the fact that the increased CD4^+^ T cells of G2/M + S phase were almost completely suppressed by sinomenine treatment through caspase 3-dependent cells apoptosis regulation (Figure 4) (Yin et al., 2007). However, aside from the cell cycle, whether sinomenine could influence the quantity, viability and proliferative capacity of CD4^+^ T cells by affecting its apoptosis or autophagy, and thus affect chronic pain, remains to be further demonstrated. In addition, sinomenine directly inhibited B cell activation through the IL4/miR-324-5p/CUE domain containing protein 2 (CUEDC2) axis, by inhibiting IKK phosphorylation and NF-kB activation (Song et al., 2015). It was also reported that sinomenine inhibited the differentiation of plasma cells (PCs) by inhibiting IL-6/JAK2/STAT2 signaling, regulated the anti-apoptotic properties of PCs, and improved the local infiltration of CD138^+^ PCs (Figure 4 Figure 5) (Liu Y. et al., 2020). However, whether sinomenine could affect chronic pain, by regulating B cell cycle, apoptosis, autophagy, polarization, or the interaction with other immune cells, remains to be further investigated.

#### 2.2.2 Sinomenine regulates the balance of Th1/Th2 and Th17/Treg cells

T cell subsets are closely related to the pathological progression of chronic pain (Sharif et al., 2018; Jiao et al., 2019; Ruterbusch et al., 2020), and the imbalance of Th1/Th2 or Th17/Treg cells plays an important role in the pathogenesis of arthritic chronic pain (Quick et al., 2013; Luchting et al., 2015; Woda et al., 2016; Motrich et al., 2020; Ding et al., 2021). Moreover, the release of interferon-gamma (IFN-γ) after Th1 cell activation could induce macrophage M1 polarization, while the secretion of IL-4 from Th2 cell could induce macrophage M2 polarization (Zhou K. et al., 2017). Several studies have shown that sinomenine regulated the balance between Th1 and Th2, as well as the Th17/Treg cells. It inhibited the serum anti-OVA IgG2a levels and IFN-γ (Th1 cell) with a dose-dependent effect, showing its analgesic effect in the adjuvant-induced arthritis model mice. The inhibitory effect was much stronger than that on serum anti-OVA IgE and IL-5 (Th2 cell) (Feng et al., 2006; Feng et al., 2007). In addition, sinomenine significantly inhibited the expression of IFN-γ secreted by Th1, reduced T-box expressed in T cells (T-bet) expression and T-bet/GATA-binding protein 3 (GATA-3) ratio, both of which were important transcription factors for Th1 and Th2 cell differentiation, suggesting that sinomenine might also achieve its inhibiting effects by interfering T cell differentiation (Luo et al., 2021).

Administration of Qing-Luo-Yin, a traditional Chinese medicine (TCM) granule within sinomenine as its main active ingredient, inhibited the phosphorylation of the c-Jun N-terminal kinases (JNK) and p65 in monocytes, reduced the differentiation of Th17 cells, and the secretion of IL-6 and IL-1β (Wang D. D. et al., 2021). Sinomenine also significantly increased CD4^+^ CD62L^+^ T cells, inhibited the proliferation of effector T (Teff) cells and the differentiation of Th1 and Th17 cells, as well as the secretion of IL-17F and IL-21, which were all reversed by aryl hydrocarbon receptor (AhR) antagonist, suggesting that sinomenine might modulate the balance between Th17 and Treg in an aryl hydrocarbon receptor (AHR)-dependent manner (Tong et al., 2016). In addition, compared to vehicle administration in CIA model, the number of Treg cells in rat intestinal tissue and serum IL-10 were both up-regulated by sinomenine treatment, while the Th17 cells and serum IL-17A were down-regulated. Together with the reduced rat joint inflammation, it suggested that sinomenine might inhibit the pathological progression of arthritis by regulating the balance of Th17 and Treg cells (Tong et al., 2015).

It is noteworthy that, the above studies mainly focused on peripheral lymphocytes, whether sinomenine regulate chronic pain by influencing the activation or the differentiation of central immune cells has not been thoroughly validated. Furthermore, sinomenine administration not only directly reduced spinal iNOS levels, the T-bet and IFN-γ expressions in the spinal cord of experimental autoimmune encephalomyelitis (EAE) rats, but also reduced the expression of iNOS from primary astrocytes (Gu et al., 2012). All these results indicated that sinomenine might be able to affect nociception and chronic pain by regulating the interaction between peripheral lymphocytes and central glial cells.

### 2.3 Effect of sinomenine on central glial cells

Due to the blood-brain/spinal barrier (BBB/BSB), peripheral immune cells are unable to enter the central nervous system. Spinal astrocytes have been shown to be closely linked to the function of sensory neurons and implicated in many types of chronic pain (Colombo and Farina 2016; Han et al., 2021), while microglia, regarded as central resident macrophages, promoting inflammation and oxidative stress, cause spinal sensitization and induce chronic pain (Kettenmann et al., 2011; O'Shea et al., 2017; Borst et al., 2021). A creasing number of studies have investigated the effects of sinomenine on central glial cells, as well as on the central sensitization. The potential mechanisms are reviewed as follows:

#### 2.3.1 Sinomenine decreases the proliferation and activation of glial cells

**FIGURE 6:**
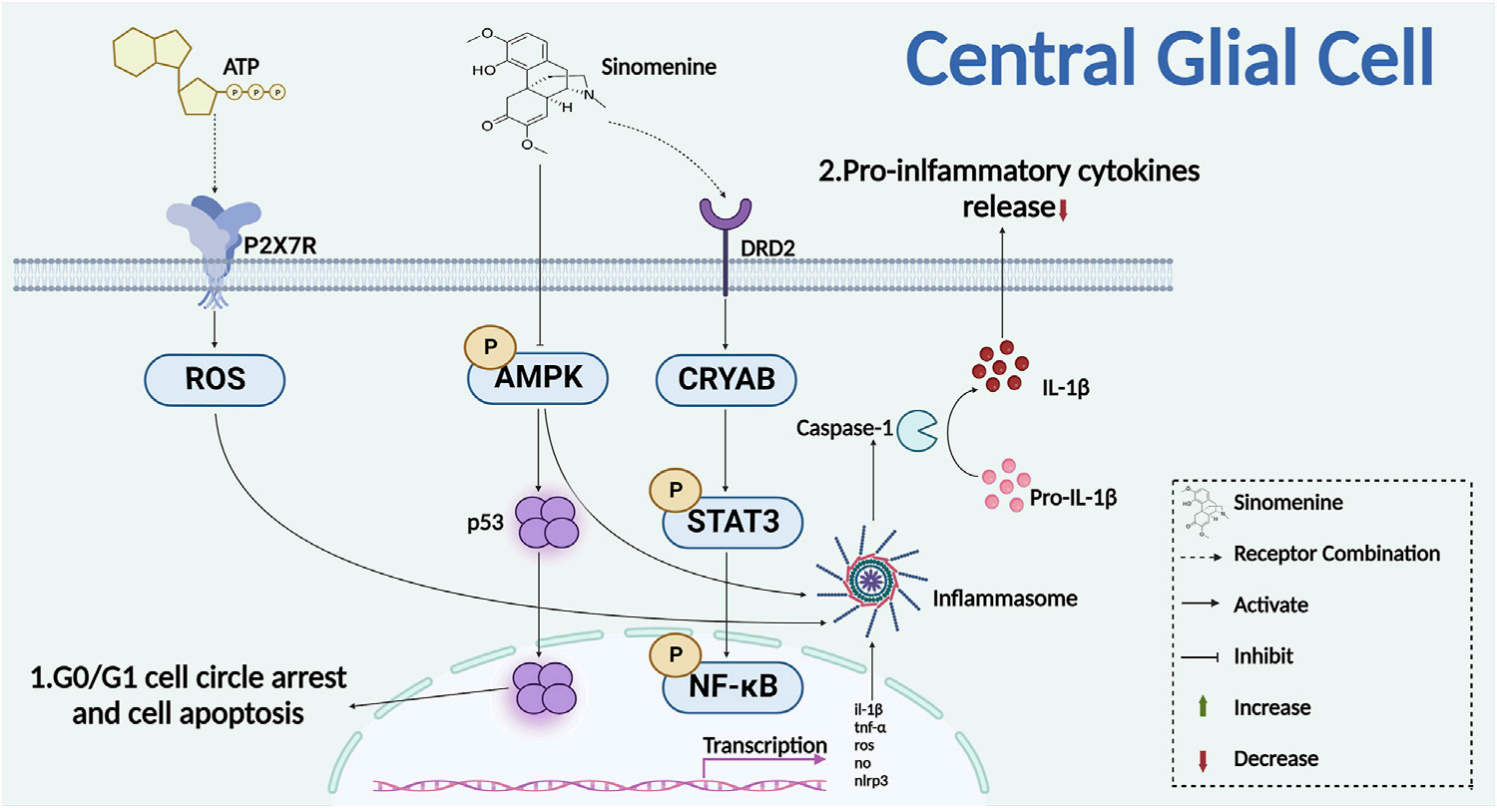
Sinomenine inhibits the proliferation of glial cells and the release of inflammatory cytokines.

Sinomenine reduced sirtuin one by promoting expression and acetylation of p53 in malignant glioma cells, then promoted G0/G1 cell cycle arrest and apoptosis, indicating that sinomenine might be able to decrease glial cell proliferation by inhibiting cell cycles and promoting apoptosis (He et al., 2018). Studies have shown that sinomenine reversed the activation of retinal microglia and BV2 cells that induced by advanced glycation end-products (AGEs) and intracerebral hemorrhage (ICH), respectively. It also significantly inhibited the release of inflammatory factors from BV2 microglia such as TNF-α, IL-1β and ROS to alleviate the neuroinflammatory injury (Wang et al., 2007; Yang et al., 2014). On the other hand, studies have reported that sinomenine administration reduced central inflammatory damage through inhibiting the activation of astrocytes, and the release of inflammatory cytokines. For example, in the middle cerebral artery occlusion (MCAO) model of mice, sinomenine enhanced the expression of the dopamine D2 receptor (DRD2) and small heat shock protein αB-crystallin (CRYAB) in astrocytes, promoted the interaction of alpha B-crystallin (CRYAB) with STAT3, and inhibited the phosphorylation of the downstream STAT3 (Figure 6). Thereby, it reduced the cytotoxic damage of neurons (Qiu et al., 2016b). It was also reported that sinomenine inhibited amyloid peptide-induced activation of astrocytes and the release of NO and ROS, showing a protective effect on neuronal injury that caused by neuroinflammation and oxidative stress after astrocyte activation (Singh et al., 2020). Furthermore, in the co-culture experiments of glial cells and primary neurons, sinomenine ameliorated the neuroinflammatory injury of neurons by inhibiting AMPK-mediated NOD-like receptor family pyrin domain containing 3 (NLRP3) inflammasome activation (Qiu et al., 2016a). Another study found that, in the multiple sclerosis (MS) model of animals, sinomenine inhibited pyroptosis degeneration of neurons by inhibiting NLRP3 inflammasome activation and Caspase-1 expression in the spinal cord, which indicated that the NLPR3 pathway of glial cells might also be one of the potential targets for sinomenine to reduce chronic pain (Kiasalari et al., 2021).

#### 2.3.2 Sinomenine inhibits microglia polarization

As central resident macrophage, the polarization of microglia is closely related to chronic pain. It was reported that, in the hippocampus of chronic constrictive sciatic nerve injury model (CCI) animals, the number of microglia with M1 polarization increased (Wang X. et al., 2021; Zhou et al., 2021). The inhibition of central microglia M1 polarization or the promotion of M2 polarization tends to be a potential way to relief chronic pain (Popiolek-Barczyk et al., 2015; Jin et al., 2020). Shi et al. found that, with pretreatment of sinomenine, the M1 polarized microglia was inhibited and the expression of microglia M2 polarization markers were elevated, suggesting one potential mechanism for sinomenine to play its analgesic effect (Shi et al., 2016). It is worth noting that the above studies did not verify the effect of sinomenine on the polarization state of microglia from a morphological and functional perspective in pain models of animals. Moreover, it remains to be further investigated whether sinomenine could exert its analgesic effect by affecting the neurotoxic or pro-inflammatory phenotype (A1), or the neuroprotective or anti-inflammatory phenotype (A2) polarization state of astrocytes (Figure 3).

## 3 Conclusions and future perspectives

Chronic pain is an intractable nervous systemic disease, which seriously threatens the patient’s quality of life. The induction of chronic pain, such as arthritic pain, has always been interpreted from the perspective of inflammation. Local inflammation increases the release of pro-inflammatory cytokines and promotes the interaction of immune and sensory cells to cause peripheral and central sensitization. In this review, we described the effects on peripheral immune cells and central glial cells of sinomenine. In addition to inhibiting pro-inflammatory cytokines release directly to achieve its immune-suppressive effects, sinomenine can also promote cell apoptosis and block the cell cycles to down-regulate the proliferation and function of immune cell subsets, and regulate the dynamic balance among different immune cells, as well as their migration and invasion.

In addition, sinomenine regulates the interactions between “immune cells,” “immune cells and neuron cells,” and “glial cells and neuron cells,” respectively: Sinomenine inhibited chronic pain by blocking the positive feedback between macrophages and CD4^+^ T cells. M1 macrophages released pro-inflammatory cytokines such as IL-1β, IL-6, and TNF-α, which could induce CD4^+^ T cells to differentiate into Th1 cells that released IFN-γ. In turn, IFN-γ induced M1 polarization of macrophages, aggravated inflammatory response, and promoted the development and maintenance of chronic pain. Similar mechanisms could also be observed in M2 polarization of macrophages and Th2 cell differentiation. Besides, sinomenine inhibited the release of autoantibodies from B cells, reducing their bindings to FcγRI on nociceptive receptors, and regulated peripheral sensitization (Feng et al., 2007; Bersellini Farinotti et al., 2019; Wang et al., 2019). Sinomenine also inhibited ROS, Maleic Dialdehyde (MDA), NF-κB and other substances released from central glial cells, and had neuron-protective effects, such as anti-oxidative stress and anti-inflammatory response, that alleviated neuronal apoptosis, central sensitization and chronic pain.

Moreover, numerous studies have shown that the activation of sensory neurons can regulate chronic pain by secreting exosomes to regulate immune cell functions. DRG neurons released extracellular vesicle-Mir-23a that taken up by macrophages, which promoted macrophage M1 polarization and exacerbated the neuroinflammatory pathology of chronic pain (Zhang et al., 2021). Increased secretion of soluble frizzled-related proteins (sFRP2) by neurons induced M1 polarization and infiltration of macrophages, and induced hyperalgesia and inflammatory responses, suggesting that regulating the interaction between neurons and macrophages might influence chronic pain (Mei et al., 2019). Similarly, Mir-21-5p released from PC12 exosomes was phagocytosed by microglia, which promoted M1 polarization of microglia, suggesting that the neurons had both effects on peripheral immune cells and central glial cells (Yin Z. et al., 2020). Although no studies have shown yet whether sinomenine has the function of interfering the mechanisms that described above, we speculate that sinomenine might play a potential role in interplaying the interaction between sensory systems and the immune cells subsets, to regulate chronic pain.

On the other hand, sinomenine was reported to alleviate abstinence reaction by suppressing the cyclic adenosine monophosphate (cAMP) level and enhancing the Cyclic guanosine monophosphate (cGMP) level in neonatal rat histaminergic neurons, evidenced by inhibiting morphine-induced conditioned place preference (CPP) in rodent models, suggesting that sinomenine might not have the side effects of addiction (Mo et al., 2005; Mo et al., 2006; Zhang et al., 2009). Sinomenine also showed its advantages of none drug resistance after repeating administration compared with opioids (Gao et al., 2014; Gao et al., 2019).

In this study, the potential analgesic mechanism of sinomenine was sorted out *via* analyzing the potential effect of sinomenine on the neuro-immune interactions. However, such as the evidences from the cecal puncture ligation (CLP), carrageenan, experimental autoimmune encephalomyelitis (EAE) and Middle cerebral artery occlusion (MCAO) relative experiments, there is a lack of previous studies with sinomenine that mediated neural-immune interaction in chronic pain. For treating chronic pain, active ingredients from traditional Chinese herb medicines might be a promising direction for the development of non-opioid-dependent analgesia. In the future, with more extensively and comprehensively basic research and clinical trials, sinomenine and its derivatives will provide a new adjuvant choice for the precise treatment of chronic pain.
